# Data Resource Profile of Shizuoka Kokuho Database (SKDB) Using Integrated Health- and Care-insurance Claims and Health Checkups: The Shizuoka Study

**DOI:** 10.2188/jea.JE20200480

**Published:** 2022-08-05

**Authors:** Eiji Nakatani, Yasuharu Tabara, Yoko Sato, Atsuko Tsuchiya, Yoshiki Miyachi

**Affiliations:** 1 Research Support Center, Shizuoka General Hospital, Shizuoka, Japan; 2 Graduate School of Public Health, Shizuoka Graduate University of Public Health, Shizuoka, Japan; 3 Center for Genomic Medicine, Kyoto University Graduate School of Medicine, Kyoto, Japan; 4 Health and Welfare Department, Shizuoka Prefectural Government, Shizuoka, Japan

**Keywords:** health checkups, Kokuho Database, Latter-Stage Elderly Medical Care System, long-term care insurance, National Health Insurance

## Abstract

**Background:**

Analyzing real-world data, including health insurance claims, may help provide insights into preventing and treating various diseases. We developed a database covering Shizuoka Prefecture (Shizuoka Kokuho Database [SKDB]) in Japan, which included individual-level linked data on health- and care-insurance claims and health checkup results.

**Methods:**

Anonymized claims data on health insurance (National Health Insurance [age <75 years] and Latter-Stage Elderly Medical Care System [age ≥75 years]), care insurance, subscriber lists, annual health checkups, and all dates of death were collected from 35 municipalities in Shizuoka Prefecture. To efficiently link claims and health checkups, unique individual IDs were assigned using a novel procedure.

**Results:**

From April 2012 to September 2018, the SKDB included 2,230,848 individuals (men, 1,019,687; 45.7%). The median age (min–max) of men and women was 60 (0–106) and 62 (0–111) years, respectively. During the study period, the median subscription time was 4.4 years; 40.8% of individuals continuously subscribed for the 6.5 years; 213,566 individuals died. Health checkup data were available for 654,035 individuals, amounting to 2,469,648 records. Care-service recipient data were available for 283,537 individuals; they used care insurance to pay for care costs.

**Conclusion:**

SKDB, a population-based longitudinal cohort, provides a comprehensive dataset covering health checkups, disorders, medication, and care service. This database may provide a robust platform to identify epidemiological problems and generate hypotheses for preventing and treating disorders in the elderly.

## INTRODUCTION

Advances in therapeutic techniques and medical technologies have contributed to health promotion and longevity. Clinical trials mostly evaluate the efficacy of new medicines, device candidates, and therapeutic regimens. However, the number of hypotheses that can be tested in a single trial is usually just one—even though a trial demands considerable time and cost. Thus, in medical heuristics, there are many potentially efficient therapies and types of care that remain unconfirmed.

Recently, individual-level real-world data, including health insurance data, have become available for medical research.^1^^–^^4^ There are residual biases and strong assumptions with the mathematical modeling of findings obtained from health insurance data^5^; however, analysis using health insurance data may be valuable in hypothesis generation and confirmation of the effectiveness of medical heuristics.^6^

In Japan, the Ministry of Health, Labour and Welfare has developed and operated the National Database of Health Insurance Claims and Specific Health Checkups of Japan (NDB) since 2008, which has been available for academic research since 2011. The NDB includes information on disease and disorder names, medical therapy details, frequency and dosage of prescribed medicines, and treatment costs.^7^^–^^10^ However, the NDB was not linked to the long-term care insurance (LTCI) database until October 1, 2020. Further, the NDB includes only individuals who used insurance to pay for medical care and underwent health checkups; the incidence and prevalence of the disease may have been incorrectly estimated owing to the lack of an identifier for all residents.

To improve the usefulness of the database, we developed a new longitudinal cohort from the Shizuoka Kokuho Database (SKDB); we did so using a unique procedure to connect individuals and remove overlaps among scattered data. The SKDB consists of data of Shizuoka Prefecture residents insured under National Health Insurance (NHI; for subjects under age 75 years) and the Latter-Stage Elderly Medical Care System (LSEMCS; age 75 years or older). The present study includes individuals who used insurance to pay for medical care, as well as all individuals insured under NHI and LSEMCS; it includes the claims of LTCI and the results of health checkups. This is a collaborative project by academia and local government. The aim of the Shizuoka Study is to clarify current and future epidemiological problems in the prefecture and identify solutions. We present here the characteristics of the SKDB.

## METHODS

### Shizuoka Kokuho Database

**Figure 1.**
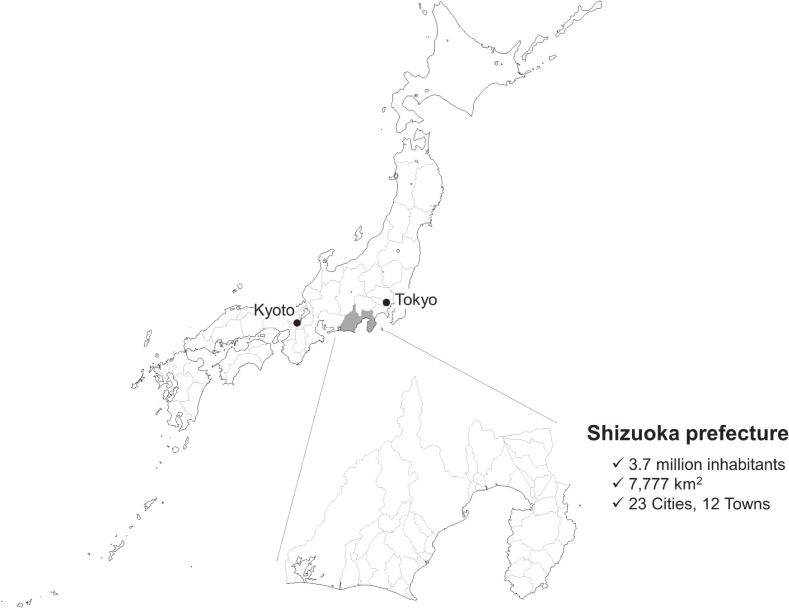
Location of Shizuoka Prefecture

Shizuoka Prefecture is located in central Japan and had 3.7 million inhabitants as of 2015 (census,^11^ Figure 1). We obtained the Kokuho Database on Shizuoka Prefecture residents from the Federation of National Health Insurance Organizations (FNHIO); it was named the SKDB. The Kokuho Database includes the monthly claims data of health insurance for NHI and LSEMCS; it also contains the results of annual health checkups and daily care-service data from LTCI for NHI and LSEMCS subscribers. All data are linked by an identifier of the Kokuho Database (KDBID). The SKDB allowed us to keep track of all subscribers, including individuals who did not use insurance to pay for medical care. Japan’s health- and care-insurance system and health checkups are explained in eMaterials 1 and eTable 1.

Variables included in the SKDB appear in Table 1. The subscriber lists included age, sex, postal code, observation period, reason for withdrawal, and death dates over the study period. The available information from the health insurance data was the patient’s disease, treatment, the care given to both outpatients and hospitalized patients, and the corresponding cost. The care-insurance data included support and care level, as well as information about care services provided for insured individuals. We also obtained the results of health checkups, which included questionnaire responses and results from laboratory examinations.

**Table 1.** Variables included in the Shizuoka Kokuho Database

Items	Major variables
**Basic Information**	
Subscriber list	Age, sex, postal code, observation period, reason for withdrawal (included all death dates during period)

**Health insurance** ^a^	
Basic information	Insurer code, hospital or pharmacy code
Disease	Disease code (ICD-compatible Japanese code), disease modifier code (eg, suspected, acute phase, atypical), ICD-10, initial treatment date
Medical receipt information	Medical receipt code for such items as laboratory tests, clinical examinations, treatment, drug dispensing; health insurance points for each medical operation; time and frequency of medical care; total medical costs
Hospitalization information	Date and duration of hospitalization

**Care insurance**	
Care levels and care services	Certified support and care level; care service code; care insurance point

**Health checkups**	
Basic information	Examination date, age, sex
Questionnaire responses	Medication, smoking, drinking history, eating, exercise, sleep habits
Anthropometric factors	Body height and weight, waist circumference (age 40–74 years)
Blood pressure	Blood pressure
Blood markers	Hematocrit, hemoglobin, erythrocyte count, aspartate aminotransferase, alanine aminotransferase, γ-glutamyl transpeptidase, triglyceride, HDL cholesterol, LDL cholesterol, fasting glucose and hemoglobin A1c, serum creatinine
Urinary markers	Urinary sugar, urinary protein
Clinical examinations	Electrocardiography, fundoscopy
Physician consultation	Disease history, medication history, subjective and objective symptoms

HDL, high-density lipoprotein; ICD, International Statistical Classification of Diseases and Related Health Problems; ICD-10, 10th revision of the ICD; LDL, low-density lipoprotein.

^a^Information on medical care and dental care are separately archived.

### Determination of unique identifiers

To analyze the SKDB, we had to prepare a unique identifier for each individual in the database, so we performed the following three procedures (eFigure 1). Problems related to the KDBID are explained in eMaterials 2. First, a new ID automatically replaces the KDBID at the time of subscribing to the LSEMCS at age 75 years; correspondence tables linking KDBIDs given for the same individuals were provided by the Shizuoka FNHIO. These lists were applied to the subscriber lists; KDBIDs were renamed as Unified KDBIDs (UKDBIDs).

Second, we excluded the following dispensable records and IDs: (1) working records generated for the insurers’ administrative purposes; (2) UKDBIDs where the sex and age information were inconsistent within the subscriber list; (3) UKDBIDs that lacked the postal code within the subscriber list; these UKDBIDs are perhaps UKDBIDs of people insured with care insurance but lacking health insurance; (4) UKDBIDs for the apparent head of a household (see eMaterials 2); (5) UKDBIDs that lacked information about subscription and withdrawal dates; and (6) UKDBIDs where the insured period fell outside the study period (April 2012 to September 2018).

Third, if there were multiple UKDBIDs with the same sex, birth date, and postal code, we selected only one. We prioritized UKDBIDs with a longer observational period with deaths and health checkups over others. The same person could not appear as multiple individuals in the SKDB.

### Determining outliers in health checkup data

To deal with outliers for laboratory data in the health checkups, we flagged values that met outlier criteria (eTable 2).^12^ When the values of height and weight or values of systolic blood pressure and diastolic blood pressure were the same, we flagged both values as outliers. In cases with three or more values, we flagged as outliers values that were ≥99.7 percentile for the standard deviation (SD) of all values (total SD) and the proportion (SD for the remaining values excluding one/total SD) was <40%, as well as values that were ≥99.9 percentile for the total SD. Those potential outliers will be reported to the individual who will conduct the statistical analysis. We also excluded multiple records (ie, records having the same UKDBID on the same measurement day).

### Statistical analysis

We analyzed the SKDB as a population-based cohort study. We prepared a longitudinal dataset based on information related to monthly claims, annual health checkups, and daily care services. In this analysis, the start date of the follow-up period was defined as the insurance registration date or April 1, 2012, whichever came first; the end date was the date of insurance withdrawal or September 31, 2018, whichever came later.

Continuous variables are presented as mean and SD or median and range; categorical variables are presented as frequency and percentage. The individuals were classified into age groups: ≤4, 5–9, 10–14, 15–19, 20–24, 25–29, 30–34, 35–39, 40–44, 45–49, 50–54, 55–59, 60–64, 65–69, 70–74, 75–79, 80–84, 85–89, 90–94, and ≥95 years; in some cases, we extended the age ranges.

We counted the numbers of cases with only NHI, both NHI and LSEMCS, and only LSEMCS. We estimated sex- and age-group-specific survival rates using the Kaplan-Meier method for the whole follow-up period. We treated withdrawals from health insurance as losses to follow-up. For our analyses, we used the age as of the initial date for the follow-up period.

We calculated the coverage rates in the SKDB against public statistics for insured individuals (on March 31, 2015, obtained from Shizuoka FNHIO) and residents (according to the census of 2015^11^) among subgroups for the insured period of fiscal 2015 (April 1, 2015 to March 31, 2016). We also determined the coverage rates for health-insurance users (with NHI and LSEMCS), care-service users (with LTCI), and individuals undergoing health checkups against the SKDB data for fiscal 2015. For these analyses, we used the age as of April 1, 2015.

We summarized the support and care levels of care insurance, using an initial certified level during fiscal 2015 for each case. We also calculated the coverage rates among care insurance users in the SKDB against residents (census of 2015^11^) among subgroups for the insured period of fiscal 2015 (April 1, 2015 to March 31, 2016).

We summarized the results of health checkups using initial results during the follow-up period for each case. We performed a summary of the results of health checkups after deleting the measurements with outlier flags.

We conducted statistical analyses using SAS version 9.4 (SAS Institute, Cary, NC, USA).

### Data disclosure

The SKDB is not accessible to the public. Researchers at certain medical institutes, such as Shizuoka General Hospital, are allowed access to the dataset for medical research following approval by the ethics committee of Shizuoka General Hospital. Owing to a contract made with Shizuoka Prefecture, local municipalities, and Shizuoka FNHIO, the SKDB can currently be accessed only by our coresearchers. From April 2021, it will be necessary to collaborate with a full-time faculty of the Shizuoka School of Public Health to access the SKDB.

### Ethical considerations

This study conforms to the Ethical Principles for Medical Research Involving Human Subjects issued by the Ministry of Health, Labour and Welfare and the Ministry of Education, Culture, Sports, Science, and Technology in Japan. We also obtained approval from each municipality review board in Shizuoka Prefecture for using the data. The Ethics Committee of Shizuoka General Hospital approved the whole research project (SGHIRB#2018058, 2018); that committee will also review individual plans to undertake additional research using the data.

Information related to this research has been disclosed on the Web sites of the FNHIO in Shizuoka Prefecture, Shizuoka Prefectural Government Office, and Shizuoka General Hospital (eTable 3). Following approval by the review committees and the information disclosure, each person’s information was anonymized and sent from the Shizuoka FNHIO to the Research Support Center of Shizuoka General Hospital for analysis.

## RESULTS

The initial subscriber list, which was provided by the Shizuoka FNHIO, included 4,499,614 KDBIDs. After applying the procedure for identifying unique IDs (eFigure 1), 2,230,848 individuals (men, 1,019,687; 45.7%) with median person-years of 4.93 (range, 0.005–6.50) years, were included in the SKDB; 910,365 cases (40.8%) continuously subscribed to health insurance over the 6.5-year period. The median age at the initial date of the study period in men and women was, respectively, 60 (range, 0–106) and 62 (range, 0–111) years, and the frequencies of the sex and age-groups classified by the year of the initial date of follow-up period appear in eTable 4. The numbers of cases with only NHI, both NHI and LSEMCS, and only LSEMCS were 1,712,297 (76.8%), 85,471 (3.8%), and 433,080 (19.4%), respectively.

**Figure 2.**
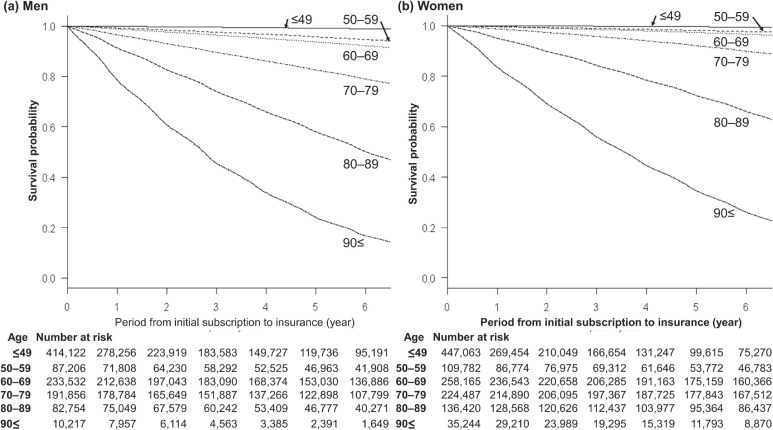
Survival probability. Age-specific survival curves for men (a) and women (b) are presented. The 5-year survival rates for men in the age-groups of <49, 50–59, 60–69, 70–79, 80–89, and ≥90 years were, respectively, 99.1%, 95.7%, 93.6%, 82.6%, 58.0%, and 24.1%; those of women were 99.5%, 98.1%, 97.2%, 92.0%, 72.3%, and 34.6%.

During the study period, among the 2,230,848 cases, the numbers of individuals who died for any reason were 110,873 (10.9%) for men and 109,323 (9.0%) for women. Figure 2 presents the age- and sex-specific survival rates from the insurers’ initial subscription to death by any cause. The sex- and age-group-specific reasons for loss to follow-up appear in Table 2.

**Table 2.** Reasons for loss to follow-up

Reason^a^	Age at last date of follow-up period					

	≤59 years		60–74 years		≥75 years	

	Men	Women	Men	Women	Men	Women

	*N* = 459,925	*N* = 503,363	*N* = 262,508	*N* = 294,065	*N* = 297,254	*N* = 413,733
Subscribed to employee health insurance	196,389 (42.7)	255,217 (50.7)	18,597 (7.1)	26,602 (9.0)		
Death	4,243 (0.9)	2,137 (0.4)	21,840 (8.3)	10,700 (3.6)	84,790 (28.5)	96,486 (23.3)
Moved to other municipality	56,880 (12.4)	61,746 (12.3)	7,970 (3.0)	8,340 (2.8)	2,089 (0.7)	4,111 (1.0)
Other	11,778 (2.6)	12,165 (2.4)	1,225 (0.5)	2,274 (0.8)	50 (0.02)	92 (0.02)
Received public assistance	5,669 (1.2)	4,095 (0.8)	3,935 (1.5)	2,379 (0.8)	1,247 (0.4)	2,147 (0.5)
Moved, merged or separated between households	4,086 (0.9)	5,294 (1.1)	365 (0.1)	808 (0.3)	—	—
Eliminated by executive of municipality for some reason	4,918 (1.1)	2,913 (0.6)	373 (0.1)	111 (0.04)	91 (0.03)	61 (0.01)
Subscribing to LSEMCS owing to age (75 years)	—	—	—	—	538 (0.2)	1,191 (0.3)
Subscribing to LSEMCS owing to disability	—	—	386 (0.1)	357 (0.1)	16 (0.01)	31 (0.01)

Individuals successfully followed up	175,962 (38.3)	159,796 (31.7)	207,817 (79.2)	242,494 (82.5)	208,433 (70.1)	309,614 (74.8)

LSEMCS, Latter-Stage Elderly Medical Care System.

^a^Reasons for loss to follow-up were obtained from the subscriber list, which included reasons for and date of withdrawal from health insurance with National Health Insurance and LSEMCS.

Categorical variables were summarized by frequency (percentage).

**Figure 3.**
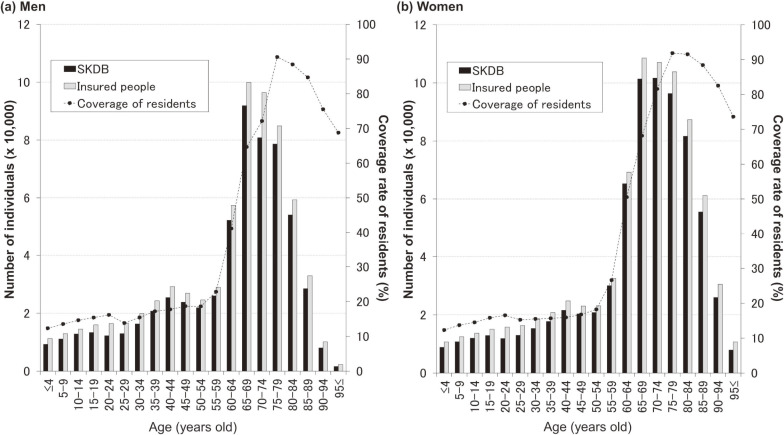
Size of the Shizuoka Kokuho Database. The bar graphs indicate the number of individuals in the Shizuoka Kokuho Database (SKDB) (black bars) or total subscribers to National Health Insurance and Latter-Stage Elderly Medical Care System (gray bars). The line graphs indicate the coverage of Shizuoka Prefecture residents by the SKDB. The values are calculated using data for fiscal 2015. Publicly available figures were obtained for the number of health insurance subscribers for fiscal 2015.

In all, 1,332,625 individuals (men, 601,750; 45.1%) were insured in fiscal 2015. The sex-specific age distribution on April 1, 2015 appears in eFigure 2; the median age among men and women was 67 (range, 0–107) and 70 (range, 0–108) years, respectively. We compared the sex- and age-group-specific case numbers with public statistics and number of residents (Figure 3). With any age-group, those numbers were close to the publicly available numbers of subscribers to NHI and LSEMCS.

**Figure 4.**
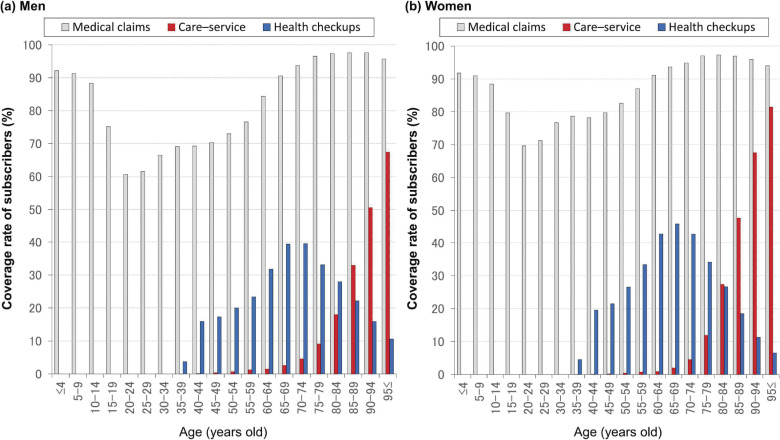
Coverage rate for health- and care-insurance users and health checkup users Utilization of health and care insurance (respectively, gray and red bars) and participation in health checkups (blue bars) was calculated using data for fiscal 2015.

The sex- and age-group-specific coverage rates for medical claims (including dental claims in NHI and LSEMCS), care-service suppliers (in LTCI), and health checkups in fiscal 2015 appear in Figure 4.

**Figure 5.**
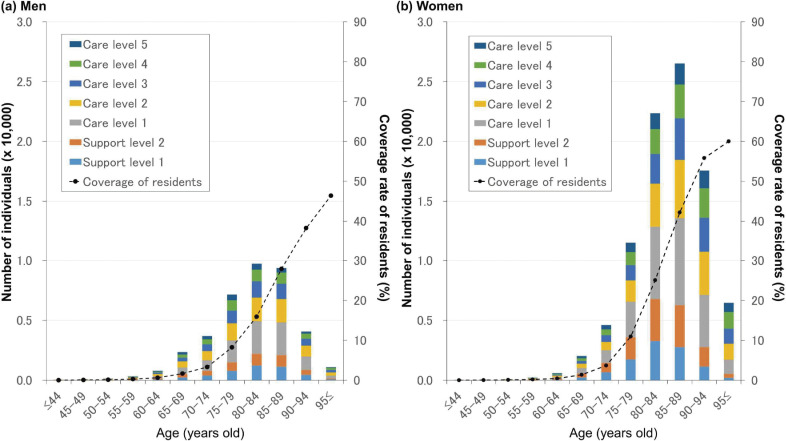
Care level–specific numbers of individuals who used care insurance to pay for care costs. We classified care-service suppliers into seven groups according to certified support and care level.

In fiscal 2015, data related to recipients of care service were available for 130,760 individuals (men, 38,807; 29.7%) who used care insurance when paying for care costs. The median age for men and women was 82 (range, 40–108) and 85 (range, 40–111) years, respectively. Figure 5 shows the frequency of age-group- and sex-specific initial care-service levels.

Health checkup data were available for 678,501 individuals (men, 288,890; 42.6%) with 2,383,523 results; those data amounted to 30.4% of the whole analysis set. Sex- and age-group-specific results of first health checkups within the study period appear in Table 3 and Table 4.

**Table 3.** Summary statistics of clinical measurements at health checkups

Variable	Unit or category	Age- at initial checkup date											

		≤59 years				60–74 years				≥75 years			

		Men		Women		Men		Women		Men		Women	

		*n* = 49,358		*n* = 67,120		*n* = 151,548		*n* = 211,008		*n* = 87,984		*n* = 111,483	

		Statistics	Missing (%)	Statistics	Missing (%)	Statistics	Missing (%)	Statistics	Missing (%)	Statistics	Missing (%)	Statistics	Missing (%)
Age	Years	49.1 (6.5)	—	50.3 (6.6)	—	66.9 (3.7)	—	67.1 (4.1)	—	79.4 (4.4)	—	80.7 (4.8)	—

Height	cm	169.5 (6.2)	42 (0.1)	156.7 (5.6)	44 (0.1)	165.1 (6)	193 (0.1)	152 (5.5)	226 (0.1)	160.8 (6.2)	148 (0.2)	146.4 (6.3)	349 (0.3)

Weight	Kg	69.4 (11.8)	120 (0.2)	54.3 (9.8)	75 (0.2)	63.4 (9.3)	231 (0.2)	51.6 (8.4)	240 (0.1)	58.6 (9.1)	131 (0.2)	47.6 (8.4)	189 (0.2)

BMI	kg/m^2^	24.1 (3.7)	84 (0.2)	22.1 (3.9)	89 (0.2)	23.2 (3)	268 (0.2)	22.3 (3.4)	342 (0.2)	22.6 (3)	207 (0.2)	22.2 (3.5)	480 (0.4)

Waist circumference	Cm	85.4 (9.9)	87 (0.2)	78.9 (10.2)	129 (0.3)	84.4 (8.3)	1,010 (0.7)	81.2 (9.7)	1,071 (0.5)	83.6 (8.7)	41,244 (46.9)	82.5 (10.1)	53,065 (47.6)

Systolic BP	mm Hg	124.6 (16.5)	57 (0.2)	118.5 (17.1)	70 (0.1)	131.5 (16.8)	264 (0.2)	129.4 (16.9)	312 (0.2)	132.5 (16.6)	93 (0.1)	134.4 (16.6)	159 (0.1)

Diastolic BP	mm Hg	78 (12.1)	77 (0.2)	71.8 (11.5)	81 (0.2)	77.7 (10.9)	284 (0.2)	74.6 (10.6)	331 (0.2)	73.6 (10.7)	92 (0.1)	72.8 (10.5)	161 (0.1)

Hematocrit	%	45.5 (3.3)	9,400 (19.0)	40.1 (3.5)	11,113 (22.5)	44.2 (3.7)	29,159 (19.2)	40.7 (3.1)	37,067 (17.6)	41.8 (4.4)	24,482 (27.8)	39 (3.8)	28,760 (25.8)

Hemoglobin	g/dL	15.1 (1.2)	9,164 (18.6)	13 (1.3)	10,877 (22.0)	14.6 (1.3)	28,897 (19.1)	13.2 (1)	36,807 (17.4)	13.6 (1.5)	24,474 (27.8)	12.5 (1.3)	28,752 (25.8)

Erythrocyte count	10^4^/µL	488.1 (41.8)	9,158 (18.6)	439.6 (35.6)	10,774 (21.8)	465.6 (44.1)	28,857 (19.0)	433.8 (36.2)	36,754 (17.4)	435.9 (49.6)	24,468 (27.8)	410.4 (42.6)	28,739 (25.8)

Triglyceride	mg/dL	148.9 (117.8)	261 (0.5)	96.1 (66.4)	80 (0.2)	127.3 (82.1)	357 (0.2)	107.6 (59.9)	225 (0.1)	110.9 (62.5)	26 (0.03)	108.6 (54.8)	17 (0.02)

HDL cholesterol	mg/dL	56.6 (15.3)	89 (0.2)	69.9 (16.8)	94 (0.2)	57.6 (15.6)	336 (0.2)	66.9 (16.2)	277 (0.1)	55.2 (15)	62 (0.1)	62.4 (15.4)	48 (0.04)

LDL cholesterol	mg/dL	126.8 (33.2)	85 (0.2)	125.9 (32.7)	101 (0.2)	121.3 (30.7)	286 (0.2)	131.5 (30.9)	452 (0.2)	112.1 (28.5)	51 (0.1)	121.2 (29.2)	106 (0.1)

AST	IU/L	25.6 (14.5)	74 (0.2)	21.3 (10.8)	58 (0.1)	25.4 (12.7)	214 (0.1)	23.6 (9)	201 (0.1)	25.3 (10.7)	82 (0.1)	24.3 (9.7)	66 (0.1)

ALT	IU/L	29.3 (21.1)	86 (0.2)	18.4 (13.6)	74 (0.2)	23.3 (14.6)	198 (0.1)	19.3 (11.5)	243 (0.1)	19.5 (11.4)	61 (0.1)	16.7 (9.8)	63 (0.1)

γ-GTP	IU/L	53.8 (66)	123 (0.3)	25.4 (29.4)	62 (0.1)	45.9 (54.8)	510 (0.3)	25.4 (24.5)	264 (0.1)	34.5 (40)	117 (0.1)	21.8 (20.3)	31 (0.03)

Fasting blood glucose	mg/dL	98.6 (24.1)	18,882 (38.3)	91.1 (15.5)	26,155 (53.0)	103 (23.2)	60,401 (39.9)	96 (17)	86,051 (40.8)	101.5 (21.2)	45,775 (52.0)	97.6 (18.3)	60,295 (54.1)

Hemoglobin A1c	%	5.6 (0.8)	742 (1.5)	5.5 (0.6)	837 (1.7)	5.8 (0.8)	1,743 (1.2)	5.7 (0.6)	1,889 (0.9)	5.8 (0.7)	3,836 (4.4)	5.7 (0.6)	4,756 (4.3)

Creatinine	mg/dL	0.8 (0.3)	3,140 (6.4)	0.6 (0.2)	4,050 (8.2)	0.9 (0.3)	10,545 (7.0)	0.6 (0.2)	13,235 (6.3)	1 (0.4)	9,161 (10.4)	0.7 (0.2)	10,581 (9.5)

eGFR	ml/min/1.73 m^2^	80 (14.9)	3,151 (6.4)	80.4 (14.9)	4,118 (8.3)	70.3 (15.1)	10,534 (7.0)	72.4 (14.6)	13,350 (6.3)	62.4 (15.7)	9,107 (10.4)	62.8 (16)	10,614 (9.5)

Uric acid	mg/dL	6.1 (1.3)	4,221 (8.6)	4.5 (1.1)	5,534 (11.2)	5.9 (1.3)	13,603 (9.0)	4.7 (1.1)	17,271 (8.2)	5.8 (1.3)	14,056 (16.0)	4.9 (1.3)	17,245 (15.5)

Urinary sugar^a^	−	46,961 (95.4)	131 (0.3)	65,955 (98.8)	374 (0.8)	141,507 (93.7)	592 (0.4)	207,032 (98.4)	666 (0.3)	81,947 (93.9)	743 (0.8)	107,345 (97.6)	1,459 (1.3)
	±	493 (1.0)		237 (0.4)		2,474 (1.6)		1,005 (0.5)		1,551 (1.8)		930 (0.8)	
	+	564 (1.1)		147 (0.2)		2,671 (1.8)		898 (0.4)		1,670 (1.9)		785 (0.7)	
	++	428 (0.9)		137 (0.2)		1,833 (1.2)		565 (0.3)		989 (1.1)		475 (0.4)	
	+++	781 (1.6)		270 (0.4)		2,471 (1.6)		842 (0.4)		1,084 (1.2)		489 (0.4)	

Urinary protein^a^	−	40,951 (83.2)	131 (0.3)	59,779 (89.6)	372 (0.8)	124,148 (82.2)	588 (0.4)	188,037 (89.4)	655 (0.3)	65,892 (75.5)	743 (0.8)	87,919 (79.9)	1,460 (1.3)
	±	5,382 (10.9)		4,995 (7.5)		15,473 (10.2)		14,953 (7.1)		10,788 (12.4)		12,263 (11.1)	
	+	2,093 (4.3)		1,567 (2.3)		7,648 (5.1)		5,591 (2.7)		6,707 (7.7)		6,950 (6.3)	
	++	583 (1.2)		310 (0.5)		2,763 (1.8)		1,429 (0.7)		2,861 (3.3)		2,259 (2.1)	
	+++	218 (0.4)		97 (0.1)		928 (0.6)		343 (0.2)		993 (1.1)		632 (0.6)	

ALT, alanine aminotransferase; AST, aspartate aminotransferase; BMI, body mass index; BP, blood pressure; γ-GTPm, γ-glutamyl transpeptidasel; HDL, high-density lipoprotein; LDL, low-density lipoprotein.

Continuous and categorical variables were summarized by means (standard deviations) and frequency (percentages). In cases with multi-year data, the earliest one was used for analysis. The estimated glomerular filtration rate (eGFR) was calculated using the following equation: 194 × creatinine^−1.094^ × age^−0.287^ (× 0.739 [if women]).

^a^The test paper was dipped into the urine sample and qualitatively classified as (−), (±), (+), (++), or (+++) according to the discoloration density of the reagent.

**Table 4.** Summary statistics for questionnaires at health checkups

Variable (Abbreviated category)	Category	Age-group at initial checkup date					

		≤59 years		60–74 years		≥75 years	

		Men	Women	Men	Women	Men	Women

		*n* = 49,358	*n* = 67,120	*n* = 151,548	*n* = 211,008	*n* = 87,984	*n* = 111,483
**Disease history**							

Stroke^a^ ​ (No)	Yes	955 (1.9)	682 (1.0)	8,050 (5.3)	5,768 (2.7)	8,064 (9.2)	6,654 (6.0)
	Missing	4,998 (10.1)	7,463 (11.1)	17,471 (11.5)	25,202 (11.9)	15,849 (18.0)	19,516 (17.5)

Coronary artery disease^b^ ​ (No)	Yes	1,146 (2.3)	751 (1.1)	11,392 (7.5)	7,905 (3.7)	11,033 (12.5)	10,917 (9.8)
	Missing	5,009 (10.1)	7,472 (11.1)	17,474 (11.5)	25,292 (12.0)	15,862 (18.0)	19,565 (17.5)

Chronic kidney disease^c^ ​ (No)	Yes	146 (0.3)	120 (0.2)	956 (0.6)	627 (0.3)	1,063 (1.2)	779 (0.7)
	Missing	5,013 (10.2)	7,476 (11.1)	17,506 (11.6)	25,262 (12.0)	15,847 (18.0)	19,499 (17.5)

Anemia^d^ ​ (No)	Yes	1,549 (3.1)	13,820 (20.6)	7,556 (5.0)	21,174 (10.0)	8,188 (9.3)	12,092 (10.8)
	Missing	5,150 (10.4)	7,701 (11.5)	18,210 (12.0)	26,256 (12.4)	15,940 (18.1)	19,742 (17.7)

**Smokers and drinkers**							

Current smoking^e^ ​ (No)	Yes	18,226 (36.9)	8,946 (13.3)	33,083 (21.8)	9,387 (4.4)	9,467 (10.8)	1,880 (1.7)
	Missing	16 (0.03)	20 (0.03)	80 (0.1)	93 (0.04)	45 (0.1)	23 (0.02)

Drinking^f^ ​ (Never)	Every day	16,364 (33.2)	7,497 (11.2)	59,114 (39.0)	12,728 (6.0)	21,379 (24.3)	2,268 (2.0)
	Sometime	12,191 (24.7)	15,686 (23.4)	30,628 (20.2)	34,074 (16.1)	13,383 (15.2)	8,996 (8.1)
	Missing	4,854 (9.8)	7,016 (10.5)	15,866 (10.5)	22,454 (10.6)	21,496 (24.4)	27,448 (24.6)

Amount of drinking^g^ ​ (<1 *go*)	1–2 *go*	10,430 (21.1)	6,982 (10.4)	38,980 (25.7)	10,215 (4.8)	13,728 (15.6)	1,582 (1.4)
	2–3 *go*	7,114 (14.4)	2,493 (3.7)	17,334 (11.4)	2,001 (0.9)	3,069 (3.5)	307 (0.3)
	>3 *go*	3,498 (7.1)	999 (1.5)	3,448 (2.3)	344 (0.2)	344 (0.4)	35 (0.03)
	Missing	10,206 (20.7)	21,216 (31.6)	33,234 (21.9)	77,952 (36.9)	33,565 (38.1)	56,552 (50.7)

**Lifestyle habits**							

≥10-kg weight gain after age 20 years^h^ ​ (No)	Yes	21,965 (44.5)	16,975 (25.3)	52,955 (34.9)	50,098 (23.7)	18,304 (20.8)	19,676 (17.6)
	Missing	2,722 (5.5)	3,562 (5.3)	9,450 (6.2)	12,713 (6.0)	16,549 (18.8)	21,786 (19.5)

≥3-kg weight change within 1 year^i^ ​ (No)	Yes	14,914 (30.2)	16,332 (24.3)	26,550 (17.5)	32,074 (15.2)	10,197 (11.6)	12,857 (11.5)
	Missing	7,223 (14.6)	10,230 (15.2)	23,785 (15.7)	33,230 (15.7)	26,850 (30.5)	34,347 (30.8)

Exercise habit^j^ ​ (No)	Yes	12,126 (24.6)	12,476 (18.6)	61,512 (40.6)	75,541 (35.8)	31,266 (35.5)	29,174 (26.2)
	Missing	5,938 (12.0)	8,550 (12.7)	20,269 (13.4)	29,254 (13.9)	24,839 (28.2)	31,901 (28.6)

Physical activity^k^ ​ (No)	Yes	20,270 (41.1)	26,012 (38.8)	75,363 (49.7)	101,847 (48.3)	40,089 (45.6)	43,551 (39.1)
	Missing	2,756 (5.6)	3,633 (5.4)	9,584 (6.3)	13,196 (6.3)	16,542 (18.8)	21,899 (19.6)

Walking speed^l^ ​ (No)	Yes	19,374 (39.3)	22,213 (33.1)	64,921 (42.8)	86,000 (40.8)	26,277 (29.9)	29,691 (26.6)
	Missing	6,038 (12.2)	8,722 (13.0)	20,799 (13.7)	30,113 (14.3)	24,990 (28.4)	32,138 (28.8)

Restorative sleep^m^ ​ (No)	Yes	30,081 (60.9)	37,980 (56.6)	105,046 (69.3)	136,487 (64.7)	51,667 (58.7)	62,224 (55.8)
	Missing	6,186 (12.5)	8,823 (13.1)	21,139 (13.9)	30,279 (14.3)	25,290 (28.7)	32,418 (29.1)

Will and effort for lifestyle habits^n^ ​ (No will)	Willing (within 6 months)	15,600 (31.6)	21,860 (32.6)	31,543 (20.8)	48,011 (22.8)	12,139 (13.8)	16,033 (14.4)
	Willing (in the near future)	7,097 (14.4)	11,544 (17.2)	16,021 (10.6)	27,883 (13.2)	5,047 (5.7)	6,814 (6.1)
	Efforts completed (<6 months)	3,794 (7.7)	5,986 (8.9)	9,919 (6.5)	16,611 (7.9)	3,327 (3.8)	4,184 (3.8)
	Efforts completed (≥6 months)	5,295 (10.7)	7,310 (10.9)	28,460 (18.8)	38,694 (18.3)	11,694 (13.3)	12,830 (11.5)
	Missing	6,145 (12.4)	8,856 (13.2)	21,192 (14.0)	31,413 (14.9)	26,097 (29.7)	34,007 (30.5)

**Eating behavior**							

Quick eating^o^ ​ (Normal)	Fast	16,599 (33.6)	15,114 (22.5)	34,060 (22.5)	37,309 (17.7)	10,531 (12.0)	10,230 (9.2)
	Slow	2,715 (5.5)	5,512 (8.2)	11,060 (7.3)	14,230 (6.7)	9,251 (10.5)	11,771 (10.6)
	Missing	5,981 (12.1)	8,631 (12.9)	20,440 (13.5)	29,298 (13.9)	24,783 (28.2)	31,773 (28.5)

Dinner within 2 h of bedtime^p^ ​ (No)	Yes	13,410 (27.2)	9,338 (13.9)	20,869 (13.8)	14,301 (6.8)	10,797 (12.3)	10,192 (9.1)
	Missing	2,731 (5.5)	3,582 (5.3)	9,504 (6.3)	12,766 (6.1)	16,501 (18.8)	21,715 (19.5)

Snacking after dinner^q^ ​ (Sometimes)	Everyday	8,513 (17.2)	10,914 (16.3)	11,782 (7.8)	16,625 (7.9)	3,827 (4.3)	4,490 (4.0)
	Missing	7,161 (14.5)	10,129 (15.1)	23,576 (15.6)	32,861 (15.6)	26,668 (30.3)	33,980 (30.5)

Skipping breakfast^r^ ​ (No)	Yes	9,712 (19.7)	7,751 (11.5)	9,017 (5.9)	7,659 (3.6)	2,264 (2.6)	2,853 (2.6)
	Missing	5,989 (12.1)	8,614 (12.8)	20,473 (13.5)	29,276 (13.9)	24,866 (28.3)	31,825 (28.5)

**Taken medication for common ailments**							

Medication for hypertension^s^ ​ (No)	Taking medicine	7,438 (15.1)	7,025 (10.5)	63,171 (41.7)	72,406 (34.3)	48,788 (55.5)	66,085 (59.3)
	Missing	16 (0.03)	21 (0.03)	72 (0.05)	89 (0.04)	30 (0.03)	8 (0.01)

Medication for hypercholesterolemia^t^ ​ (No)	Taking medicine	4,072 (8.2)	5,473 (8.2)	28,943 (19.1)	62,175 (29.5)	19,145 (21.8)	38,808 (34.8)
	Missing	16 (0.03)	23 (0.03)	79 (0.1)	95 (0.05)	33 (0.04)	9 (0.01)

Medication for diabetes^u^ ​ (No)	Taking medicine	2,191 (4.4)	1,346 (2.0)	16,553 (10.9)	12,334 (5.8)	10,881 (12.4)	9,175 (8.2)
	Missing	17 (0.03)	25 (0.04)	76 (0.1)	97 (0.05)	32 (0.04)	8 (0.01)

Categorical variables were summarized by frequency (percentage). The questionnaires at the health checkups addressed the following questions; ^a^Have you been told by a physician that you have suffered a stroke (eg, cerebral hemorrhage, cerebral infarction) or have you ever received treatment for stroke? ^b^Have you been told by a physician that you suffer from heart disease (eg, angina pectoris, myocardial infarction) or have you ever received treatment for heart disease? ^c^Have you been told by a physician that you suffer from chronic renal failure or have you ever received treatment for chronic renal failure (dialysis)? ^d^Have you been told by a physician that you suffer from anemia? ^e^Are you currently a habitual smoker? ^f^How often do you drink alcoholic beverages (eg, sake, distilled spirit, beer, whiskey, wine)? ^g^How much do you drink a day? The amount of drinking was assessed using the Japanese liquor unit of *go*, whereby 1 *go* corresponds to 22 g of ethanol. ^h^Has your body weight increased by 10 kg or more since age 20? ^i^Have you undergone a weight gain or loss of 3 kg or more in the past year? ^j^Have you performed exercise that involved slight sweating for 30 minutes or more at least twice a week for over 1 year? ^k^Do you walk or engage in some physical exercise equivalent to walking for 1 hour or more a day? ^l^Do you walk faster than people who are of around the same age as you and the same sex? ^m^Do you sleep well and get sufficient rest? ^n^Do you want to improve your life habits regarding eating and exercise? ^o^Do you eat faster than other people? ^p^Do you eat dinner within 2 hours before sleeping at least three times a week? ^q^Do you eat any snacks after dinner (a bedtime snack other than three regular meals) three times or more a week? ^r^Do you miss breakfast three times or more a week? ^s^Are you taking medication to reduce your blood pressure? ^t^Are you taking medication to reduce your cholesterol level? ^u^Are you taking insulin injections or medication to reduce your blood sugar?

## DISCUSSION

The SKDB is a prefecture-wide, individual-level linked, and longitudinal dataset; it comprises health- and care-insurance claims and the results of health checkups. Several studies have used the Kokuho Database of a single municipality^13^ and annual-linked data^14^; however, the SKDB is the first prefecture-wide, longitudinal dataset, and it includes over 2 million Shizuoka Prefecture residents. Several studies using the SKDB have been reported.^15^^–^^17^ In the future, the SKDB will be updated by adding further data, starting from October 1, 2018.

Health insurance data have become widely used for epidemiological studies in several countries. The Taiwan National Health Insurance Research Database is the largest nationwide population database; it includes approximately 23 million Taiwanese,^1^ and by 2018 over 2,700 medical papers related to it had been published.^18^ The Netherlands, Scandinavian countries, and South Korea have also established national health-insurance databases.^2^^–^^4^ In Japan, the NDB has covered almost all health-insurance claims submitted electronically from medical institutions since 2009.^19^^,^^20^ The NDB covers only individuals who used insurance to pay for medical care and undertook health checkups; it does not include all insured individuals. Furthermore, the NDB was not linked to the LTCI database until October 1, 2020. Accordingly, compared with other countries, health-insurance data in Japan have not been fully utilized for epidemiological studies. Accordingly, we believe that even though the SKDB covers a single prefecture, not the whole of Japan, it has complete personal links between insurance claims data and health checkup data; thus, it is appropriate for epidemiological analysis in several ways.

The coverage of the SKDB for individuals aged <75 years is limited to residents who enrolled in the NHI; it does not include people who subscribed to employee health insurance. However, all individuals ≥75 years are included in the SKDB. Japan is becoming a super-aged society ahead of other parts of the world^21^^,^^22^; thus, population-based data about older people constitutes essential information for understanding and addressing related problems. For other developed countries facing a rapidly aging population, it is essential to understand such health problems as frailty and to identify solutions. The SKDB may be one of the prime options for providing health-care evidence for an older Asian population.

The Japan Medical Data Center (JMDC) has a health-insurance claims dataset that can be used for medical research.^23^^,^^24^ That dataset mainly includes individual-level linked data on health-insurance claims and specific health checkups; the data were obtained mainly from insurers receiving employee health insurance. The SKDB, however, does not cover beneficiaries from employee health insurance. The JMDC dataset does not include data on retired people; it is unsuitable for analyzing geriatric diseases (such as bone fractures, dementia, and terminal care) and long-term care. The possibility to undertake such analyses is a strength of the SKDB. Accordingly, JMDC data and organized Kokuho Databases, such as the SKDB, may need to complement one another.

An extensive database makes it possible to grasp the basic epidemiology in certain diseases with low incidence, including rare conditions. For example, the incidence of progressive multifocal leukoencephalopathy among patients with autoimmune diseases was determined by analyzing a United States health insurance database.^25^ Kuo et al assessed familial aggregation of systemic lupus erythematosus and other autoimmune diseases from the data of over 18,000 patients.^26^ Using the SKDB as an extensive dataset may help determine the characteristics (eg, lifestyle information from health checkups) of several low-incidence diseases in addition to calculating the incidence and prevalence. The SKDB may be useful for finding solutions to health-care issues that cannot easily be assessed by hospital- and population-based cohorts.

It is difficult to precisely conduct a prognosis analysis (including an economic analysis) of cancer patients. In the future (as of November 18, 2020), if it becomes possible to match the population-based cancer registry data in Shizuoka Prefecture^27^ with the SKDB, the detailed baseline characteristics of cancer and detailed cause-of-death information will be added to the SKDB. Conversely, the SKDB provides a population-based cancer registry with medical or long-term care services, as well as more detailed medical information, such as comorbidities in cancer patients. Thus, a precise analysis, which is currently not possible, may be conducted in future by combining the SKDB with other data.

### Limitations

When analyzing the SKDB, there are several limitations regarding identifiers, care-insurance claims, health checkups, and cause of death. First, with respect to identifiers, we found cases with multiple KDBIDs by checking the coincidence of sex, birth date, and postal code such that only a single ID remained in the SKDB. Different people having exactly the same information could be deleted over suspicion of being the same person. Second, conversely, cases with multiple KDBIDs may not be eliminated; for example, individuals who moved to another municipality in Shizuoka Prefecture within the study period may have had different KDBIDs and thus were treated as different people. Third, we treated readmission cases with the same KDBID as continuous subscribers; however, data on insurance claims and specific health checkups during a period of temporary withdrawal were not available.

Regarding care-insurance claims, first, only two digits—not six digits—of the care-service code were available, so we lacked details about that service, knowing only approximately the type of long-term care service. Thus, a detailed analysis of care services provided for insured individuals is impractical using the SKDB. Second, we could not analyze all insured individuals receiving LTCI: the dataset included claims data only for care-service receivers who used care insurance to pay for care costs. Further, certified information about care levels was unavailable. The SKDB included only about 84% of care-service receivers from March to September 2018 among the individuals certified as needing care services in March 2018^28^; thus, individuals with poorer health and with a certified care level could not be accurately identified.

Health checkups are not mandatory for older people in Japan; systems for health checkups depend on the annual health administration policy of each municipality. Therefore, in a subpopulation with available health checkups results, there may be subgroups that should be distinguished by year and municipality in addition to sex and age. We investigated the presence of associations among those four classification variables, as well as among other variables; perhaps owing to the large number of cases, almost all the tests for independence were significant (*P* < 0.001, data not shown). Thus, to avoid a severe bias when assessing health checkup data, the classification variables should be confirmed and an adjustment analysis should be undertaken when analyzing the SKDB.

This database contains all deaths and dates of death (provided by the FNHIO), but the cause of death is unknown. It may be identifiable by extracting the disease name code for the months before death.

### Conclusion

The SKDB is organized as an individual-level linked, population-based longitudinal cohort; it comprises data of Shizuoka Prefecture residents with NHI and LSEMCS. The dataset covers all individuals insured with NHI and LSEMCS, not just those receiving medical care. The database also has the results of subscriber lists, health checkups, LTCI claims data, and data with all death dates. The SKDB may be useful for addressing health-care issues of older people and epidemiological issues unclarified using conventional population-based cohorts.

## SUPPLEMENTARY MATERIAL

eFigure 1. Flow for producing the analysis-ready subscriber list in the Shizuoka Kokuho Database. KDBID, identifier of Kokuho Database; LSEMCS, Latter-Stage Elderly Medical Care System; NHI, National Health Insurance; UKDBID, Unified KDBID. ^a^The time to submit the information differed depending on each municipality; however, by March 31, 2012 at the latest, the Shizuoka FNHIO obtained each insured person’s transfer information (joining, withdrawal, moving in, and moving out) for all municipalities in Shizuoka Prefecture. That information was used as the basis for the subscribers’ information files. Since April 2012, the Shizuoka FNHIO has received monthly reports from the municipalities (including transfer information and details of additional insured individuals) and added those data to the subscribers’ information files. In all, 4,499,614 KDBIDs had been included in the subscribers’ information files by January 28, 2019 (after September 31, 2018) obtained from the Shizuoka FNHIO.

eFigure 2. Age distribution of the Shizuoka Kokuho Database population

Age was calculated as of April 1, 2015 for individuals insured from April 1, 2015 to March 31, 2016

eMaterials 1. Japanese health- and care-insurance system and health checkups

eMaterials 2. Problems using KDBID as an identifier

eTable 1. Health- and care-insurance systems in Japan

eTable 2. Acceptable range of clinical measurements

eTable 3. URLs for information disclosure

eTable 4. Number of individuals for sex- and age-groups classified by years at initial date of follow-up period.
