# Supplementary material for: Data Resource Profile of Shizuoka Kokuho Database (SKDB) Using Integrated Health- and Care-insurance Claims and Health Checkups: The Shizuoka Study
Source: J Epidemiol. 2022 Aug 5;32(8):391–400. doi: 10.2188/jea.JE20200480 (PMC9263618; doi:10.2188/jea.JE20200480)
Supplement: Supplementary file 1 [file je-32-391-s001.pdf]

Initial list

4,499,614 KDBIDs (identifiers of Kokuho Database)[a]

- New KDBID issued when an individual was re-subscribed to LSEMCS at the age of 75 and original KDBID for NHI were merged into one UKDBID. — 296,546

4,203,068 UKDBIDs (Unified KDBIDs)

- 1) Working records — 16,361
- 2) Inconsistency in sex or birthday on some records with the same UKDBID — 401
- 3) Lack of postal code — 247,703
  (including IDs with care insurance but not health insurance data)
- 4) Possible cases of constructive householders — 389,532
- 5) Lack of information on enrollment and withdrawal date — 59,036
- 6) Follow-up period is outside from April 2012 to September 2018 — 1,076,300

Technically qualified list

2,413,735 UKDBIDs

- Possible cases of multiple individuals — 182,887

Analysis-ready subscriber list

2,230,848 individuals

eFigure 1. Flow for producing the analysis-ready subscriber list in the Shizuoka Kokuho Database. KDBID, identifier of Kokuho Database; LSEMCS, Latter-Stage Elderly Medical Care System; NHI, National Health Insurance; UKDBID, Unified KDBID. [a] The time to submit the information differed depending on each municipality; however, by March 31, 2012 at the latest, the Shizuoka FNHIO obtained each insured person's transfer information (joining, withdrawal, moving in, and moving out) for all municipalities in Shizuoka Prefecture. That information was used as the basis for the subscribers' information files. Since April 2012, the Shizuoka FNHIO has received monthly reports from the municipalities (including transfer information and details of additional insured individuals) and added those data to the subscribers' information files. In all, 4,499,614 KDBIDs had been included in the subscribers' information files by January 28, 2019 (after September 31, 2018) obtained from the Shizuoka FNHIO.

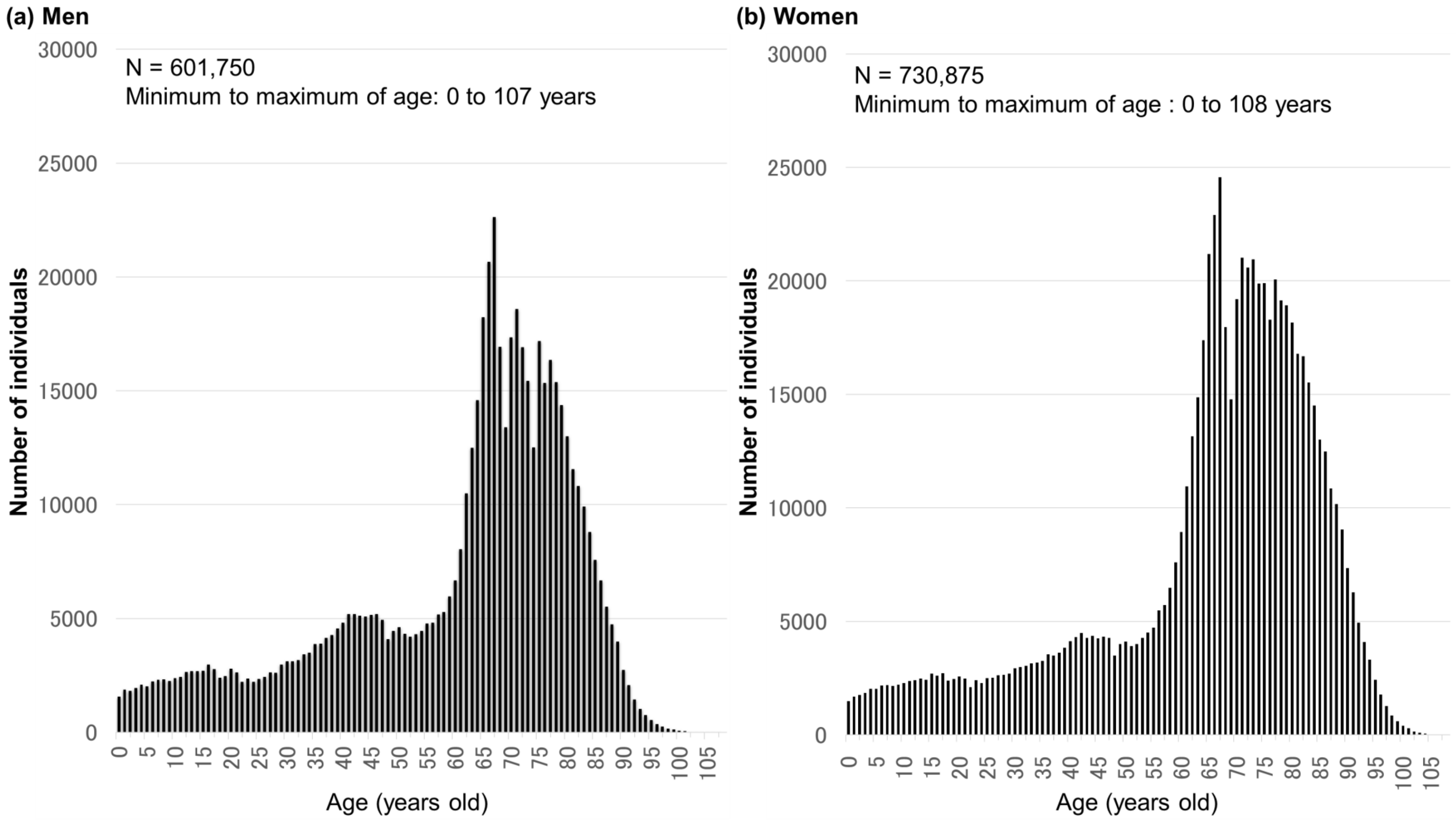

eFigure 2. Age distribution of the Shizuoka Kokuho Database population

Age was calculated as of April 1, 2015 for individuals insured from April 1, 2015 to March 31, 2016

eMaterials 1. Japanese health- and care-insurance system and health checkups

Overview of Japanese health- and care-insurance system

Japan's medical system is characterized by universal insurance: all residents of Japan have a health insurance plan (**eTable 1**).[1] In Japan, there are two major types of health insurance for people aged <75 years. One is employee health insurance for people working in companies, public service personnel, private school teachers, and their families. The other is National Health Insurance (NHI) for small-business employees, sole proprietors who do not work for companies or organizations, and primary industry workers (such as in agriculture and fisheries) and their families. Those who retire without continuing their voluntary health insurance payments become enrolled in NHI. Health insurance for people aged ≥75 years and those aged 65–75 years and certified as having specific disabilities are covered by Latter-Stage Elderly Medical Care System (LSEMCS).

For care insurance, there is long-term care insurance (LTCI) for people aged ≥40 years (**eTable 1**). Insured individuals are divided into primary insured (≥65 years) and secondary insured people (40–64 years) based on differences in requirements for receiving and collecting insurance. Thus, Japan's lifetime medical system comprises health and care insurance.

Health insurance system

Japan achieved universal health coverage for medical care in 1961 by establishing employee-based and community-based social health insurance systems (**eTable 1**). Individuals working for large companies have to enroll in the company-based health insurance. Employees in small to medium-sized companies that lack a company-based health insurance system have to enroll in NHI, which is operated by the Japan Health Insurance Association. NHI is designed for individuals who are ineligible to qualify for employee health insurance, e.g., people working in agriculture and fisheries, self-employed, and people out of work. Individuals who withdraw from employee health insurance through retirement also enroll in NHI. When NHI subscribers reach age 75 years, they are automatically registered to the LSEMCS.

Any health insurance in Japan guarantees freedom of choice of medical institution (free access). In general, when an insured person or household member receives medical care at a hospital or clinic, ≥70% of the medical expenses are covered by insurance.

Care insurance system

LTCI (**eTable 1**) is designed to cover daily care expenses, particularly with older people. Insured individuals who need long-term care can receive several services (e.g., home-visit

nursing care, home-visit rehabilitation, nursing home short stays, in-home medical care management counseling); they can also receive services at long-term care welfare facilities for the elderly or other facilities according to their certified care level. When insured individuals apply to their municipality to receive care service, the long-term care approval board determines whether they should receive support level (1–2) or care level (1–5) according to the mental and physical conditions reported by the applicant and their doctor's opinion. Long-term care requirement certification is designed for uniform, objective application nationwide.

Health checkup programs

The Act on Assurance of Medical Care for Elderly People[2] obliges all subscribers aged 40–74 years to undergo annual health checkups (*tokutei kenshin*). Standard items in the health checkups are listed in **Table 1**. With NHI subscribers in Shizuoka Prefecture, the overall participation rate in health checkups in fiscal 2015 was 52.9%. The act also stipulates that efforts should be made for health checkups (*koki-koreisha kenshin*) to be undertaken by people aged ≥75 years who subscribe to the LSEMCS; however, the health checkups are not mandatory. Those health checkups generally cater to subscribers aged over 40 years.

References

1. Ikegami N, Yoo BK, Hashimoto H, et al. Japanese universal health coverage: evolution, achievements, and challenges. *Lancet*. Sep 2011;378(9796):1106-15.

2. Act on Assurance of Medical Care for Elderly People. Ministry of Health, Labour and Welfare. https://elaws.e-gov.go.jp/search/elawsSearch/elaws_search/lsg0500/detail?lawId=357AC0000000080; Accessed 05.09.2020.

eMaterials 2. Problems using KDBID as an identifier

Data on health and care insurance and health checkups were provided to the central Federation of National Health Insurance Organizations (FNHIO) from the Shizuoka FNHIO. The central FNHIO then gave a unique ID (KDBID) to each subscriber using IDs for insured people as individual identifiers. It is possible for multiple KDBIDs to be assigned to the same person in the following cases: e.g. (1) people who re-enrolled in NHI after withdrawal owing to temporary residence in another municipality; (2) people who were temporarily enrolled in employee health insurance; (3) people aged 75 years during the study period and had a new KDBID with LSEMCS and an old KDBID with NHI; and (4) people who moved to another municipality in Shizuoka Prefecture within the study period. Further, it is possible that KDBIDs may be assigned to people without NHI membership.

The head of a household is obliged to pay the tax in a household that includes a person covered by National Health Insurance; however, some heads of households are not insured with National Health Insurance. Such heads of households are termed apparent heads of households and are not covered by National Health Insurance, although they had to be registered in the database for insurance business and had a KDBID.

eTable 1. Health- and care-insurance systems in Japan

Insurance system	Insurer (no.)	Insured individuals	Age (years)	Number (million)
**Health insurance**				
National Health Insurance	Cities, towns, and villages (1,880)	Non-subscribers to employee health insurance[a]	<75	35
Employee health insurance	Health insurance societies (1,405)	Employees in large companies[a]	<75	29
	Japan Health Insurance Association (1)	Employees in small to medium-sized companies[a]	<75	37
	Mutual aid associations (85)	Public service personnel[a]	<75	9
Medical care system for the latter-stage elderly	Association of Latter-Stage Elderly Healthcare (47)	Individuals aged 75 years or older[b]	≥75	16
**Care insurance**				
Long-term Care Insurance	Cities, towns, and villages (1,571)	Primary insured people	≥65	34
		Secondary insured people	40–64	42

[a]Including family members

[b]Including individuals aged 65 years or older confirmed as having a designated level of disability

The numbers of insurers and insured individuals are as of fiscal 2017.

eTable 2. Acceptable range of clinical measurements

Variable, unit	Acceptable range	
	Minimum	Maximum
Body height, cm[a]	100.0	250.0
Body weight, kg[a]	20.0	250.0
BMI, kg/m^2[a]	10.0	100.0
Waist circumference, cm[a]	40.0	250.0
Systolic BP, mmHg[a]	60	300
Diastolic BP, mmHg[a]	30	150
Hematocrit, %[a]	0.0	100.0
Hemoglobin, g/dL[a]	0.0	30.0
Erythrocyte count, 10^4/μL[a]	0	1000
Aspartate aminotransferase, IU/L[a]	0	1000
Alanine aminotransferase, IU/L[a]	0	1000
γ-glutamyl transpeptidase, IU/L[a]	0	1000
Triglyceride, mg/dL[a]	10	2000
HDL cholesterol, mg/dL[a]	10	500
LDL cholesterol, mg/dL[a]	20	1000
Fasting blood glucose, mg/dL[a]	20	600
Hemoglobin A1c, %[a]	3.0	20.0
Creatinine, mg/dL	0.1	20
eGFR, ml/min/1.73 m^2	1	500

BMI, body mass index; BP, blood pressure; eGFR, estimated glomerular filtration rate; HDL, high-density lipoprotein; LDL, low-density lipoprotein.

[a]Values were obtained from specific health checkups or health guidance programs from the Ministry of Health, Labour and Welfare, Japan

eTable 3. URLs for information disclosure

Organization	URL
Shizuoka Federation of National Health Insurance Organizations	https://www.shizukokuhoren.or.jp/rinshokokai/
Shizuoka Prefecture	http://www.pref.shizuoka.jp/kousei/ko-030a/studyboard.html
Shizuoka General Hospital	http://www.shizuoka-pho.jp/sogo/section/21_5ac56b85584c1/clinical-trial/info-disclosure/index.html

eTable 4. Number of individuals for sex- and age-groups classified by years at initial date of follow-up period.

Age,[a] years	Year of initial date of follow-up period													
	2012		2013		2014		2015		2016		2017		2018	
	Men	Women	Men	Women	Men	Women	Men	Women	Men	Women	Men	Women	Men	Women
≤4	17,044	16,280	5,625	5,331	4,907	4,645	4,258	4,303	3,922	3,904	3,450	3,316	2,429	2,300
5–9	16,139	15,549	2,706	2,547	2,144	2,080	1,852	1,714	1,574	1,528	1,377	1,330	1,003	923
10–14	17,854	16,854	2,437	2,309	1,851	1,789	1,615	1,519	1,262	1,288	1,180	1,050	868	817
15–19	18,939	18,189	3,228	3,251	2,602	2,646	2,293	2,325	2,095	2,121	1,959	1,924	1,468	1,481
20–24	21,539	22,601	6,031	7,825	4,998	6,370	4,627	5,827	4,400	5,211	4,557	5,202	3,617	4,047
25–29	22,962	25,495	6,281	9,059	5,107	7,558	4,520	6,711	4,116	5,840	3,918	5,296	2,982	3,805
30–34	26,186	25,664	5,250	7,319	4,243	6,206	3,633	5,324	3,287	4,633	3,179	4,156	2,219	2,916
35–39	31,901	29,151	4,295	5,891	3,607	4,957	3,074	4,300	2,694	3,779	2,498	3,294	1,717	2,341
40–44	32,464	28,609	3,770	4,976	3,261	4,404	2,829	3,705	2,550	3,350	2,339	3,056	1,645	2,170
45–49	26,539	24,544	2,866	3,926	2,567	3,546	2,311	3,134	2,009	2,923	1,990	2,659	1,463	1,970
50–54	27,851	27,479	2,637	3,869	2,168	3,388	1,998	2,937	1,727	2,667	1,602	2,424	1,209	1,864
55–59	35,648	42,070	2,914	5,227	2,422	4,681	2,176	4,096	1,871	3,586	1,709	3,182	1,274	2,312
60–64	81,434	94,379	8,631	8,848	6,729	8,146	5,742	7,040	4,755	6,208	4,553	5,520	3,629	4,184
65–69	87,038	94,929	5,777	5,197	5,617	5,245	5,362	5,221	5,243	4,982	5,143	4,784	3,879	3,482
70–74	56,332	92,744	1,533	2,647	1,527	2,515	1,484	2,235	1,377	1,872	1,447	1,909	1,192	1,529
75–79	86,547	101,129	9,918	3,634	8,757	3,285	8,123	3,304	6,464	2,978	4,684	2,791	2,471	1,915
80–84	54,352	79,572	104	241	128	296	142	247	133	229	134	236	87	190

85–89	27,240	54,363	71	191	82	176	67	195	60	158	76	194	78	132
90–94	7,888	25,725	31	100	22	79	27	90	31	95	35	90	26	74
95≤	2,123	8,852	5	17	8	18	3	23	8	27	6	28	4	26

[a]Age was calculated at initial date of follow-up period.
